# Inhibition of Arachidonate 12/15-Lipoxygenase Improves α-Galactosidase Efficacy in iPSC-Derived Cardiomyocytes from Fabry Patients

**DOI:** 10.3390/ijms19051480

**Published:** 2018-05-16

**Authors:** Yueh Chien, Shih-Jie Chou, Yuh-Lih Chang, Hsin-Bang Leu, Yi-Ping Yang, Ping-Hsing Tsai, Ying-Hsiu Lai, Kuan-Hsuan Chen, Wei-Chao Chang, Shih-Hsien Sung, Wen-Chung Yu

**Affiliations:** 1 Institute of Pharmacology, School of Medicine, National Yang-Ming University, Taipei 11217, Taiwan; g39005005@gmail.com (Y.C.); ohyeahchou@gmail.com (S.-J.C.); ylchang@vghtpe.gov.tw (Y.-L.C.); 2 Department of Medical Research, Taipei Veterans General Hospital, Taipei 11217, Taiwan; molly0103@gmail.com (Y.-P.Y.); figatsai@gmail.com (P.-H.T.); d49405004@gmail.com (Y.-H.L.); 3 Department of Pharmacology, Taipei Veterans General Hospital, Taipei 11217, Taiwan; sharkshine@gmail.com; 4 Institute of Clinical Medicine, School of Medicine, National Yang-Ming University, Taipei 11217, Taiwan; hsinbangleu@gmail.com (H.-B.L.); mr.sungsh@gmail.com (S.-H.S.); 5 Heath Care and Management Center, Taipei Veterans General Hospital, Taipei 11217, Taiwan; 6 Division of Cardiology, Department of Medicine, Taipei Veterans General Hospital, Taipei 11217, Taiwan; 7 Center for Molecular Medicine, China Medical University Hospital, Taichung 40447, Taiwan; T21443@mail.cmuh.org.tw

**Keywords:** Fabry cardiomyopathy, iPSC, enzyme replacement therapy, Alox12/15

## Abstract

(1) Background: A high incidence of intervening sequence (IVS)4+919 G>A mutation with later-onset cardiac phenotype have been reported in a majority of Taiwan Fabry cohorts. Some evidence indicated that conventional biomarkers failed to predict the long-term progression and therapeutic outcome; (2) Methods: In this study, we constructed an induced pluripotent stem cell (iPSC)-based platform from Fabry cardiomyopathy (FC) patients carrying IVS4+919 G>A mutation to screen for potential targets that may help the conventional treatment; (3) Results: The FC-patient-derived iPSC-differentiated cardiomyocytes (FC-iPSC-CMs) carried an expected IVS4+919 G>A genetic mutation and recapitulated several FC characteristics, including low α-galactosidase A enzyme activity and cellular hypertrophy. The proteomic analysis revealed that arachidonate 12/15-lipoxygenase (Alox12/15) was the most highly upregulated marker in FC-iPSC-CMs, and the metabolites of Alox12/15, 12(S)- and 15(S)-hydroxyeicosatetraenoic acid (HETE), were also elevated in the culture media. Late administration of Alox12/15 pharmacological inhibitor LOXBlock-1 combined with α-galactosidase, but not α-galactosidase alone, effectively reduced cardiomyocyte hypertrophy, the secretion of 12(S)- and 15(S)-HETE and the upregulation of fibrotic markers at the late phase of FC; (4) Conclusions: Our study demonstrates that cardiac Alox12/15 and circulating 12(S)-HETE/15(S)-HETE are involved in the pathogenesis of FC with IVS4+919 G>A mutation.

## 1. Introduction

Fabry disease is an X-linked recessive lysosomal storage disorder that results from a deficiency of α-galactosidase A (GLA) [1,2]. Fabry cardiomyopathy (FC) is known as the major highly prevalent Fabry disease-associated morbidity [3]. Left ventricular hypertrophy (LVH), the most common presentation of FC as a result of the progressive intracellular accumulation of globotriaosylceramide (Gb3), is potentially alleviated by early enzyme replacement therapy (ERT) with GLA [4]. For the populations of Fabry cohorts in Taiwan, a majority of Fabry patients have been identified to carry GLA IVS4+919 G>A mutation and late-onset cardiac phenotype at high incidence [5,6,7,8,9,10]. Clinical trials have demonstrated that ERT can reduce the risk of major clinical events, remodel the left ventricle, improve cardiac function, and increase exercise tolerance [11] However, disease progression still occurs in a minority of FC patients, particularly those with myocardial fibrosis after ERT [11]. LysoGb3 has been used as a Fabry disease-specific marker. However, certain reports have indicated that lysoGb3 or Gb3 might not be suitable biomarkers for monitoring the long-term progression of FC and therapeutic outcome of ERT, including in Fabry patients carrying IVS4+919 G>A mutation [10,12,13]. To date, the natural course of this cardiac variant of Fabry disease and suitable biomarkers for the long-term monitoring of its progression remains mostly unclear. Improvement of conventional therapeutic regimen is therefore urgently needed, especially in ERT-insensitive FC patients carrying IVS4+919 G>A mutation.

Induced pluripotent stem cell (iPSC) technology holds promising potential in stem cell research and regenerative medicine. Recent evidences have demonstrated that, patient- or disease-specific iPSCs are feasible for modeling disease phenotypes [14]. Patient iPSC-derived CMs recapitulate various pathophysiological features and exhibit a promising potential for disease modeling, including long-QT syndrome [15,16], arrhythmogenic right ventricular dysplasia [17], and hypertrophy cardiomyopathy [18]. Notably, an iPSC-CM-based drug screening system could serve as an industry-standard preclinical screening platform to identify the effects of well-characterized drugs and elucidate the underlying risk factors of drug-induced side effects [19]. Interestingly, cellular reprogramming technology using patient-specific iPSCs has also provided an opportunity to solve the limitations for investigating inherited lysosomal storage disorders. Meng et al. generated Fabry-iPSCs from a mouse model of lysosomal storage diseases [20]. Kawagoe et al. recently generated human Fabry-iPSCs using the Sendai virus vector, and showed Gb3 accumulation in Fabry-iPSCs [21]. These findings highlight the potential utility of patient-specific iPSCs and suggest that it would be feasible to use FC-specific iPSCs (FC-iPSCs) or FC-iPSC-differentiated CM-like cells (FC-iPSC-CMs) as a platform for disease modeling, biomarker identification, and drug screening.

Mass spectrometry-based proteomics is an effective approach for performing global investigations of proteome profiles in stem cell biology and cardiovascular research [6,7,22,23]. Previous research has used Fabry disease-derived plasma or urine samples for proteomic analysis and identification of new markers of Fabry disease [24,25,26]. However, not much of proteomic analysis was performed on the clinical samples of cardiac biopsy or CM cultures for identification of the cardiac-specific biomarkers in FC. In this study, we generated FC-specific iPSC-CMs (FC-iPSC-CMs) from FC patients carrying IVS4+919 G>A mutation and screened them for the most upregulated factor in FC-iPSC-CMs using liquid chromatography–mass spectrometry and proteomic analysis. We further investigated the involvement of such factors in the progression of FC under the treatment of ERT drug in the FC-iPSC-CM platform in vitro. Targeting such a target may improve the conventional therapeutic regimen in ERT-insensitive FC patients carrying IVS4+919 G>A mutation.

## 2. Results

### 2.1. Oct4/Sox2/Nanog/Glis1-Mediated Reprogramming of Fabry Patient-Derived Somatic Cells into iPSCs

**Figure 1:**
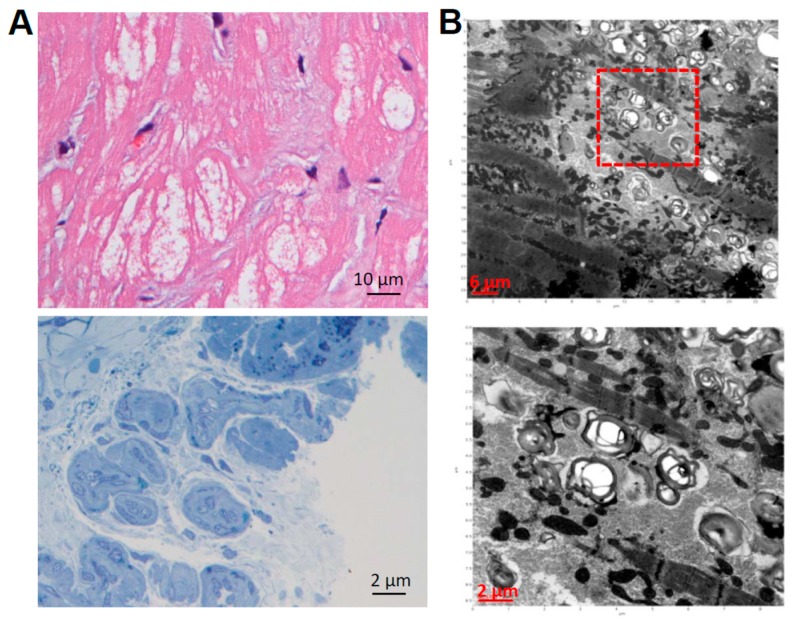

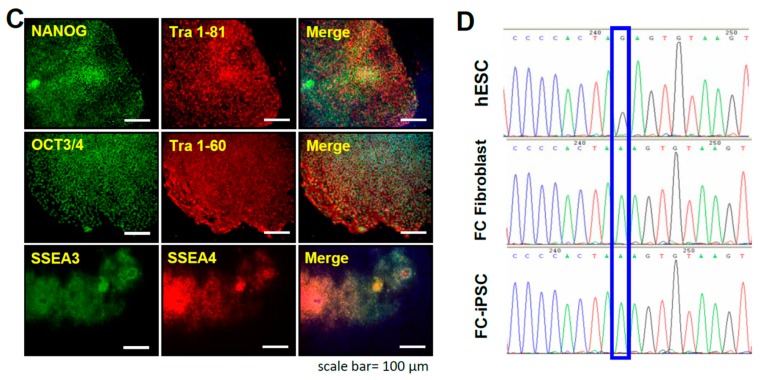
Generation of FC-iPSCs. (**A**) Patients who carry intervening sequence (IVS) +919 G>A mutation and marked ventricular hypertrophy were assigned for myocardial biopsy. In the biopsied myocardial specimen, H & E staining showed the obvious hypertrophy and disorganization of cardiomyocytes containing large perinuclear sarcoplasmic vacuoles (**upper**). The toluidine blue staining verified the accumulation of glycosphingolipid in the myocardium (**lower**); (**B**) Transmission electron microscope (TEM) examination detected the formation of lamellar bodies (Zebra bodies) that represented glycolipid-containing lysosomes × 60,000 (**lower**). Red square indicated the abnormal lysosomes containing glycolipids. H & E staining, toluidine blue staining, and TEM examination (**A**,**B**) are representative results from a patient with IVS4+919 GLA G>A mutation and cardiomyopathy; (**C**) Immunofluorescence indicated the pluripotency markers Oct3/4, Nanog, SSEA3, SSEA4, TRA-1-60 and TRA-1-81 in FC-specific iPSCs (FC-iPSCs) clones. Scale bar, 100 μm; (**D**) Sequence analysis confirmed the existence of the specific IVS4+919 G>A mutation in patient-specific FC-iPSCs. Blue rectangle indicated the G>A mutation detected by sequence analysis. Results indicated the representative IVS4+919 G>A mutation in a patient-derived FC-iPSC clone.

Cellular reprogramming technology and patient-specific iPSCs provide an opportunity to overcome the current limitations in investigating inherited lysosomal storage disorders. To investigate the disease mechanisms involved in Fabry cardiomyopathy, ten patients were enrolled in the initial study (7 males and 3 females, median age: 60.5 years; range: 40–71 years) who were diagnosed with late-onset Fabry cardiomyopathy (FC) and assigned for myocardial biopsy. In the myocardial biopsied specimens, H & E staining indicated the obvious hypertrophy and disarray of cardiomyocytes containing perinuclear sarcoplasmic vacuoles (Figure 1A, upper), and the toluidine blue staining verified the accumulation of glycosphingolipid in the myocardium (Figure 1A, lower). Transmission electron microscopy (TEM) further detected the formation of lamellar bodies (Zebra bodies) that represented glycolipid-containing lysosomes (Figure 1B, upper and lower). To generate FC-iPSCs, skin fibroblasts from ten Fabry patients with the IVS4+919 G>A mutation were obtained for the iPSC generation. Because Glis1 enhances the reprogramming efficiency of iPSCs along with the conventional factors Oct4/Sox2/Klf4 [27,28], we used Oct4/Sox2/Klf4/Glis1 combination to generate the FC-iPSCs. The skin-derived fibroblasts were transduced with a retroviral vector encoding Oct4/Sox2/Klf4/Glis1. Subsequently, these cells were re-plated onto mitotically inactivated MEFs one week after transfection [29] and were ready for the iPSC colony selection three week post-transfection. At least three individual patient-derived iPSC lines were generated per patient and assessed their pluripotency using various tests. Control iPSCs were simultaneously generated from control subjects. All established Ctrl-iPSCs and FC-iPSCs consistently expressed stemness genes including Oct4, Sox2, Nanog, SSEA-3, SSEA-4, Trai-1-60, and Trai-81 (Figure 1C). For the genomic DNA sequencing analysis, we analyzed and compared the DNA sequence among human embryonic stem cell line H9 (hESC), Fabry cardiomyopathy patient-derived skin fibroblasts (FC fibroblasts), and the patient-specific iPSCs (FC-iPSCs; reprogrammed from FC fibroblasts). The single nucleotide mutation IVS4+919 GLA G>A were detected in both FC fibroblasts and FC-iPSCs. hESCs were used as the normal control human cells carrying the normal G single nucleotide without mutation at intron IVS4+919 (Figure 1D). These findings indicated that FC-iPSCs that generated from FC patients with IVS4+919 G>A mutation consistently carried the IVS4+919 G>A mutation (Figure 1D). These FC-iPSCs were further assigned for cardiac induction and subsequent experiments.

### 2.2. Recapitulation of Cardiac Hypotrophy and GLA Enzymatic Decrease in FC Patient-Specific iPSC-Derived Cardiomyocytes (FC-iPSC-CMs)

**Figure 2:**
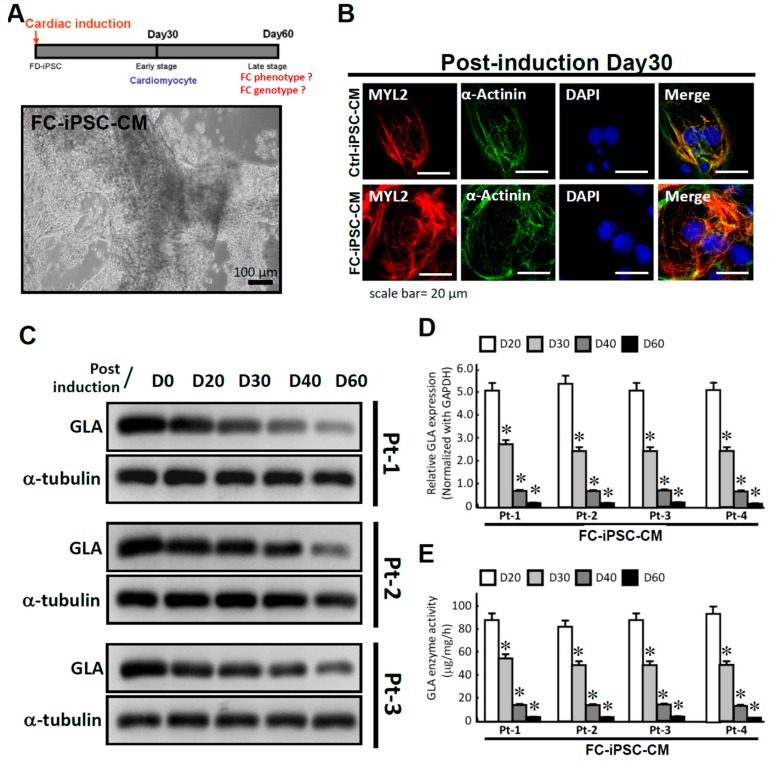
Differentiation of FC-iPSC-CMs. (**A**) Cardiac-specific differentiation protocol for FC-iPSC-CMs (**upper**). Phase-contrast photomicrograph of synchronized beating FC-iPSC-CMs (**lower**); (**B**) Immunofluorescence for cardiac markers α-actinin and MYL2 in FC-iPSC-CMs at post-differentiation day 14. The scale bar is 15 μm; (**C**) Western blot analysis of GLA protein levels in FC-iPSC-CM during the differentiation course; (**D**) GLA enzyme assay showed the decreased GLA enzyme activity in FC-iPSC-CMs during differentiation course (Pt1 to Pt4). In (**D**), the results are mean ± S.D. of three independent experiments. * *p* < 0.05 vs. D20 in corresponding FC-iPSC-CM clone.

Previous study demonstrated that patient-derived iPSCs chronologically exhibit typical characteristics of cardiomyopathy, such as familial hypertrophic cardiomyopathy (FHC), after a defined period of cardiac differentiation [30]. We sought to investigate whether FC-iPSC-CMs also chronologically recapitulate the pathophysiological characteristics of Fabry-specific cardiomyopathy. Ctrl and FC-iPSCs with established pluripotent features were further assigned for cardiac induction. After the cardiac differentiation, these differentiated cardiomyocytes (FC-iPSC-CMs) all exhibited CM-like phenotypes (Figure 2A) and the expression of cardiac-specific markers, including α-actinin and MYL2, at post-induction 30 days (Figure 2B). Comparing with Ctrl-iPSC-CMs, the microscopic examination of all FC-iPSC-CMs revealed remarkable cellular hypertrophy, as described previously [31,32]. Moreover, during differentiation, the mRNA and protein expression levels of GLA (Figure 2C,D), as well as the enzyme activity of GLA (Figure 2E), were all declined and reached the maximal decrease at day 60 post-induction. Collectively, these data indicated to the recapitulation of cardiac hypertrophy and GLA enzymatic decrease in patient-derived FC-iPSC-CMs.

### 2.3. Upregulation of Cardiac Alox12/15 and Its Secretory Metabolites 12(S)-HETE and 15(S)-HETE in FC-iPSC-CMs

**Figure 3:**
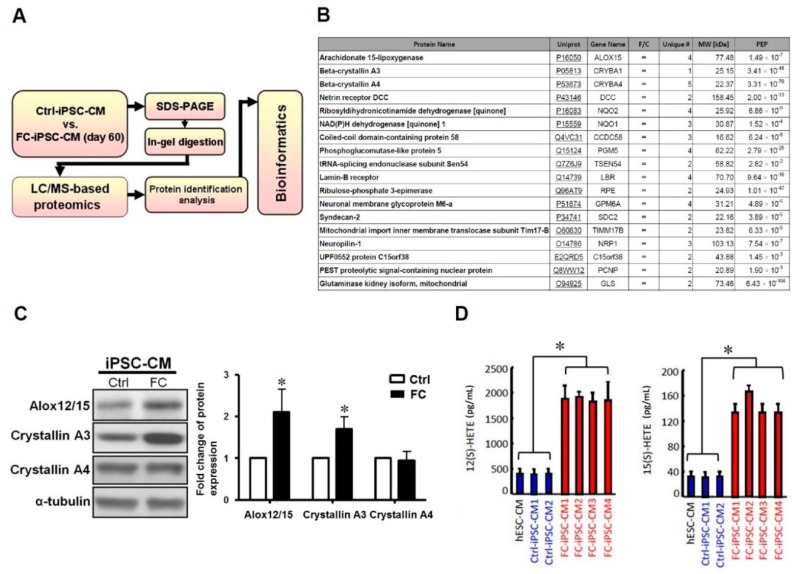
Alox12/15 as the most upregulated factor in FC-iPSC-CMs. (**A**) Schematic diagram of the MS-based proteomics, showing how candidate markers were identified in FC-iPSC-CMs. (**B**) Proteome screening of the significantly upregulated candidate markers in FC-iPSC-CMs. (**C**) Western blotting (**left**) and its quantification (**right**) confirmed the upregulation of Alox12/15 in FC-iPSC-CMs. (**D**) Elevated secretion of 12(S)-HETE and 15(S)-HETE from FC-iPSC-CMs. In (**C**,**D**), the results are mean ± S.D. of three independent experiments. In (**C**), * *p* < 0.05 vs. Ctrl; in (**D**), * *p* < 0.05 vs. Ctrl-iPSC-CM and hESC-CM.

The recapitulation of FC-specific characteristics in FC-iPSC-CMs indicated that they may serve as a useful in vitro disease model for investigating the pathogenesis of FC and screening for potential FC-specific biomarkers. Here, we used liquid chromatography–mass spectrometry-based proteomic analysis (LC/MS) and bioinformatics to screen for suitable markers of FC in patient-derived FC-iPSC-CMs (Figure 3A). Among the identified proteins, arachidonate lipoxygenases 12/15 (Alox12/15) were the most highly upregulated in FC-iPSC-CMs as compared with Ctrl-iPSC-CMs (Figure 3B). The increased Alox12/15 in FC-iPSC-CMs was also confirmed by Western blot (Figure 3C). Enzyme-linked immunosorbent assay (ELISA) analysis revealed that the secretion of Alox12/15 metabolites, 12(S)-HETE and 15(S)-HETE, was also increased in the culture medium of FC-iPSC-CMs (Figure 3D).

**Figure 4:**
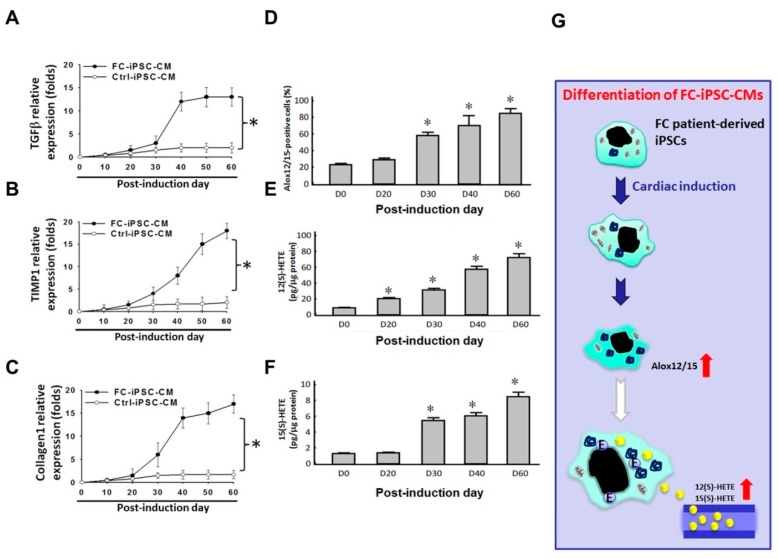
Elevation of fibrotic markers and the secretion of 12(S)-HETE and 15(S)-HETE during the differentiation course. mRNA expression levels of (**A**) TGFβ, (**B**) TIMP-1, and (**C**) collagen 1 in FC-iPSC-CMs and Ctrl-iPSC-CM at indicated time points after the induction of myocardial differentiation. (**D**) Immunofluorescence indicated the increased amount of Alox12/15-positive cells during the induction course. Elevated secretion of (**E**) 12(S)-HETE and (**F**) 15(S)-HETE from FC-iPSC-CMs. (**G**) The Scheme showing the upregulation of Alox12/15 and elevated secretion of its metabolites during the differentiation course. Red up arrows indicated the elevated levels of Alox12/15 and its metabolites. In (**A**–**E**), the results are mean ± S.D. of three independent experiments. In (**A**–**C**), * *p* < 0.05 vs. D60 in Ctrl-iPSC-CMs; in (**D**–**F**), * *p* < 0.05 vs. D0, D: Day.

Myocardial fibrosis in FC is a progressive process that cannot be reversed by ERT and is a crucial outcome determinant [33,34,35]. In addition, previous studies have proposed a role of Alox12/15 and its metabolites in the development of cardiac fibrosis and systolic dysfunction [36,37]. Therefore, we sought to investigate the correlation between expression of Alox12/15 and fibrotic markers (i.e., collagen 1, transforming growth factor β (TGFβ), and tissue inhibitor of metalloproteinase-1 (TIMP-1)). Using quantitative RT-PCR, we first observed upregulation of fibrotic markers (collagen1, TGFβ, and TIMP-1) at day 40 of differentiation, which reached the maximal expression by day 60 (Figure 4A–C). Immunofluorescence revealed that Alox12/15 protein was increased in a time-dependent manner during the differentiation time course (Figure 4D). ELISA showed that the secretion of 12(S)-HETE and 15(S)-HETE was also elevated in a time-dependent manner (Figure 4E,F). The expression patterns of Alox12/15 and its metabolites 12(S)-HETE and 15(S)-HETE during the course of differentiation exhibited a similar pattern to those of fibrotic markers. To summarize, we compared the differential protein expression profiles between control FC-iPSC-CMs and identified that cardiac Alox12/15 and its metabolites 12(S)-HETE and 15(S)-HETE might be involved in the pathogenesis of Fabry-associated cardiomyopathy (Figure 4G).

### 2.4. Time Dependency of α-Galactosidase Treatment Efficacy on Cardiomyocyte Hypertrophy in FC-iPSC-CMs

**Figure 5:**
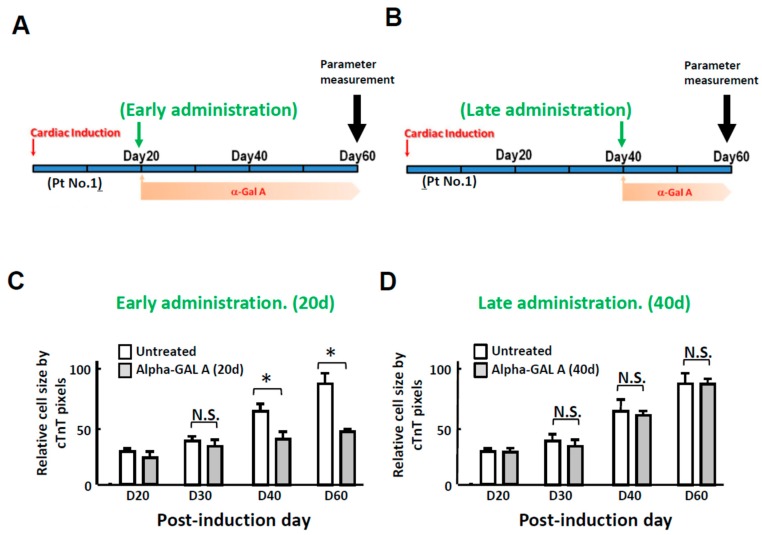
Late administration of α-galactosidase failed to ameliorate cardiomyocyte hypertrophy and upregulation of fibrosis markers. (**A**) Scheme depicting the experimental design of early administration and (**B**) late administration of ERT in the FC-iPSC-CM platform. Time-course analysis of the relative size of FC-iPSC-CM cells receiving (**C**) early-administration and (**D**) late-administration of α-galactosidase or vehicle. In (**C**,**D**), the results are mean ± S.D. of three independent experiments. * *p* < 0.05 vs. Untreated control, D: Day; N.S.: Not significant.

Since our results have demonstrated that FC-iPSC-CMs mimic FC-associated cardiac manifestations at days 40 to 60 of differentiation (Figure 2), we therefore examined the treatment effect of continuous α-galactosidase (5 μg/mL) administration beginning from day 20 post-induction (at this stage FC-iPSC-CMs still exhibit no abnormal phenotypes; Figure 5A) and from day 40 post-induction (at this stage FC-iPSC-CMs exhibit most of FC-like cardiac manifestations; Figure 5B). Quantification of c-TnT-positive margins indicated that the cardiomyocyte hypertrophy in FC-iPSC-CMs was prominently reduced by early administration of α-galactosidase (Figure 5C), but not by late administration (Figure 5D). Our findings revealed that late administration of α-galactosidase failed to ameliorate cardiomyocyte hypertrophy.

### 2.5. Simultaneous Alox12/15 Inhibition Improves the Treatment Efficacy of Late Administration of α-Galactosidase in FC-iPSC-CMs

**Figure 6:**
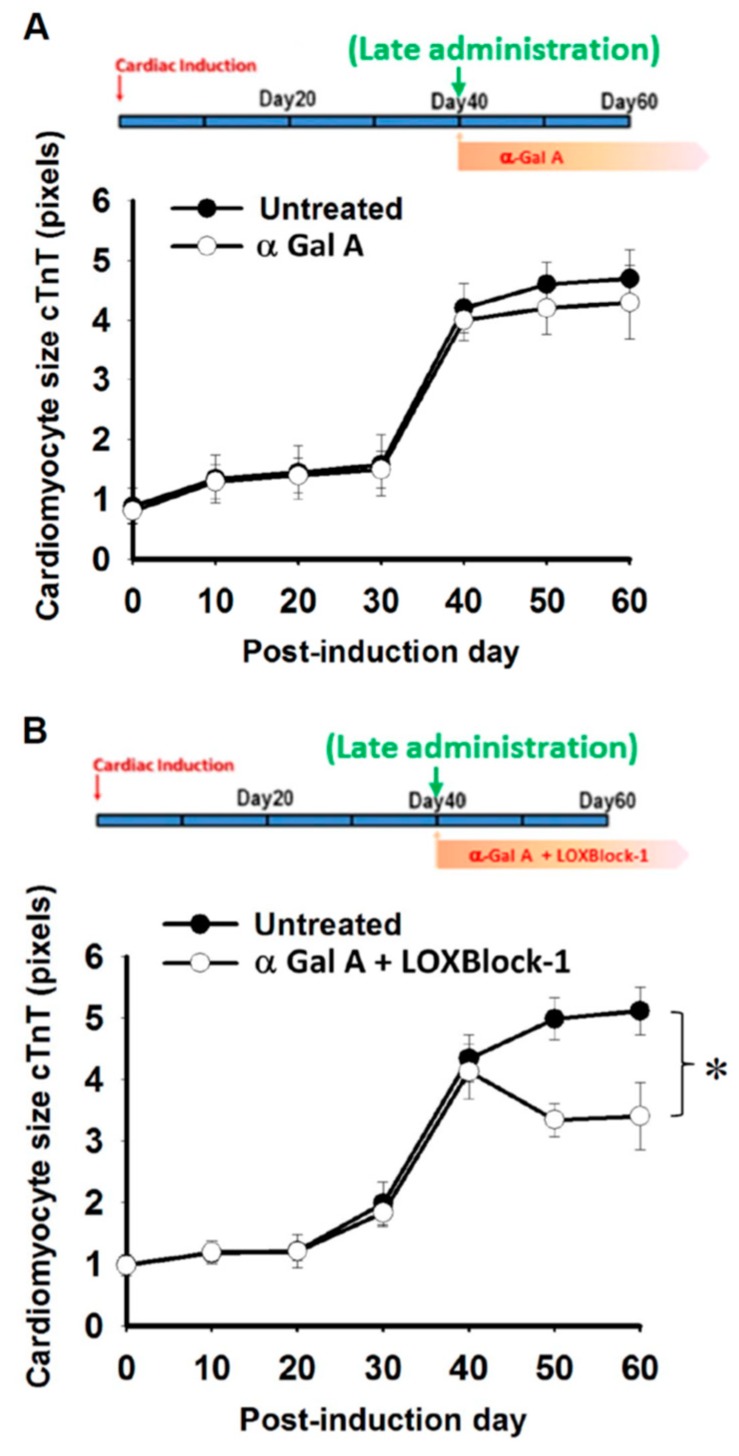
Late administration of α-galactosidase plus Alox12/15 pharmacological inhibitor ameliorates cardiomyocyte hypertrophy. cTnT-positive FC-iPSC-CMs was quantified to evaluate the cell size of FC-iPSC-CM receiving (**A**) late-administration of either α-galactosidase alone or (**B**) a combination of GLA and LOXBlock-1. In (**A**,**B**), the results are mean ± S.D. of three independent experiments. * *p* < 0.05 vs. Untreated control at D60.

**Figure 7:**
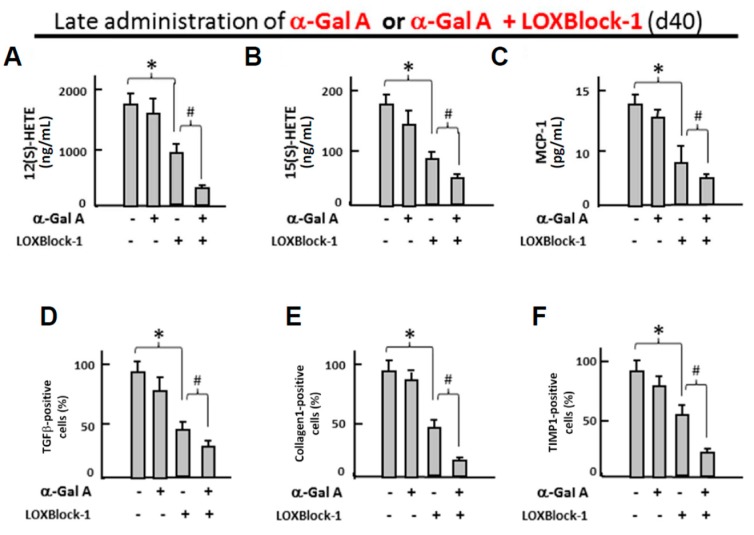
Late administration of α-galactosidase plus Alox12/15 pharmacological inhibitor suppresses the metabolites of Alox12/15, and the expression of MCP-1 and fibrosis markers. Effect of late administration of GLA or a combination of GLA and LOXBlock-1 on I, the secretion of (**A**) 12(S)-HETE, (**B**) 15(S)-HETE, and the relative expression ratio of (**C**) MCP-1 (**D**) TGFβ, (**E**) collagen-1, and (**F**) TIMP-1 in FC-iPSC-CMs. In (**A**–**F**), the results are mean ± S.D. of three independent experiments. * *p* < 0.05 vs. untreated FC-iPSC-CMs. # *p* < 0.05 vs. LOXBlock-1 alone.

Late administration of enzyme replacement therapy drugs has been known to be less effective for the treatment of Fabry patients [33], and it remains unknown whether targeting Alox12/15 can serve as an adjacent therapy for the conventional ERT treatment of FC. To test this hypothesis, FC-iPSC-CMs with low GLA expression/activity and cardiomyocyte hypertrophy were treated with α-galactosidase (5 μg/mL) at day 40 post-induction in the presence or absence of 2 µM LOXBlock-1, an Alox12/15 inhibitor (Figure 6A,B). Late administration of α-galactosidase showed negligible effect on cardiomyocyte hypertrophy (Figure 6A), despite the inhibition of LysoGb3 secretion (Supplemental Figure S1). Compared with late administration of α-galactosidase alone (Figure 6A), the addition of 2 µM LOXBlock-1, potently enhanced the effect of late-administered α-galactosidase and reduced the cardiomyocyte size (Figure 6B). Late administration of α-galactosidase alone also did not ameliorate the secretion of 12(S)-HETE and 15(S)-HETE, monocyte chemo-attractant protein-1 (Figure 7A–C, respectively), as well as the upregulated fibrotic markers collagen 1, TGFβ, and TIMP-1 (Figure 7D–F, respectively). Remarkably, the combination of LOXBlock-1 and α-galactosidase effectively suppressed the secretion of 12(S)-HETE, 15(S)-HETE, MCP-1 and the upregulation of fibrotic markers collagen 1, TGFβ, and TIMP-1. Taken together, our data indicated that targeting Alox12/15-related pathways may serve as an adjacent treatment that improves the poor efficacy of α-galactosidase in FC-iPSC-CMs with phenotypic abnormalities.

## 3. Discussion

The progress in iPSC technology has brought promising potential in regenerative medicine and personalized therapy. Remarkably, comparing with embryonic stem cells, iPSCs also carry several advantages, including less immune rejection and the lack of any ethical issue. iPSCs are capable of differentiation into several lineages and specialized cells including cardiomyocytes. Patient-derived iPSC-differentiated cardiomyocytes (iPSC-CMs) have been reported to recapitulate various disease features and serve as an excellent platform for disease modeling, for example long QT syndrome [15,16], hypertrophy cardiomyopathy [18] and others. Importantly, patient-specific iPSCs also provide the opportunity for the investigation of the pathophysiology and disease progression of inherited rare diseases, such as Fabry disease. For example, Eto Y. et al. have used a Sendai virus-based technology to generate Fabry-iPSCs, which exhibited typical features of Fabry disease [21]. Iter et al. further reported the effective clearance of Gb3 in FC-iPSC-CMs by substrate reduction therapy [38]. In Taiwan Fabry cohorts with high incidence of IVS4+919 G>A mutation and later-onset cardiac phenotype, the mechanisms that contribute to development of FC in such cardiac variant remained poorly understood. We previously demonstrated that neutralization of interleukin-18 effectively ameliorated the cardiomyocyte hypertrophy in FC-iPSC-CMs from Fabry patients with IVS4+919 G>A mutation [32]. Chou et al. further reported that conventional ERT did not modify the impaired energy utilization in FC-iPSC-CMs carrying IVS4+919 G>A mutation [31]. In the present study, we also generated FC-iPSC-CMs and adopted such a human in vitro system for modeling FC with IVS4+919 G>A mutation. These FC-iPSC-CMs chronologically recapitulated both pheno- and genotypes of FC, including the poor responsiveness to α-galactosidase after a long period of induction. We used liquid chromatography–mass spectrometry and proteomic analysis and identified Alox12/15 as the most highly upregulated factors in FC-iPSC-CMs with IVS4+919 G>A mutation and terminal differentiation. Inhibition of Alox12/15 improved the efficacy of α-galactosidase that effectively ameliorated severe cardiomyocyte hypertrophy in such a cellular model.

Lipoxygenases are a group of closely related dioxygenases that are classified as 5-, 12-, and 12/15-lipoxygenases according to the site of oxygen insertion within arachidonic acid, which is also a target for other enzymatic pathways, including cyclooxygenase (COX2), and cytochrome P450 [39,40,41]. Alox12/15 modulates the inflammatory response and is involved in atherosclerosis [42]. Overexpression of Alox12/15 in mice promotes monocyte–endothelial cell interactions, leading to atherosclerotic formation [43,44]. In the heart, cardiac Alox12/15 has been reported to stimulate cardiac cell growth [45] and is involved in cardiac dysfunction and fibrosis in diabetic cardiomyopathy [37]. A recent study further showed that cardiac fibrosis was increased in Alox12/15 transgenic mice and was associated with the infiltration of macrophages, indicating its role in the pathogenesis of inflammation and fibrosis in heart failure [36]. TGFβ has been known to serve as a key mediator of fibrosis in various organs [46]. TIMP-1 promotes tissue fibrosis by inhibiting extracellular matrix degradation and has been used as a marker of fibrosis [47]. In the present study, our findings demonstrated Alox12/15 as the most upregulated factor in FC-iPSC-CMs carrying IVS4+919 G>A mutation. Meanwhile, these FC-iPSC-CMs chronologically exhibited several FC-specific features, downregulation of GLA, upregulation of TGFβ, TIMP-1, collagen-1 and Alox12/15, after a 40 to 60-day cardiac induction. The expression pattern of Alox12/15 was highly correlated with severe cardiomyocyte hypertrophy and several fibrosis-associated factors TGFβ, TIMP-1, and collagen-1 are upregulated in the late phase of cardiac induction. These data suggested that Alox12/15 may play a role in the pathogenesis and/or disease progression of FC with IVS4+919 G>A mutation.

Studies using pharmacological inhibitors or gene abrogation have indicated that the Alox12/15 pathway can be a potential therapeutic target in several disease models, such as oxidative stress-induced neuronal cell death, focal ischemia [48], and osteoporosis [49]. In the cardiovascular system, the disruption of cardiac Alox12/15 attenuates atherosclerosis in apo E-deficient mice [50,51] and attenuates cardiac inflammation in mice with severe transverse aortic constriction [36] and diabetic cardiomyopathy [37]. With respect to the potential of cardiac Alox12/15 as a therapeutic target in FC, our FC-iPSC-CMs in vitro pharmaceutical platform showed that a combination of late-administered ERT and Alox12/15 inhibitor LOXBlock-1 synergistically reduced the secretion of 12(S)- and 15(S)-HETE, the cardiomyocyte hypertrophy, and suppressed the upregulation of fibrotic markers in FC-iPSC-CMs (Figure 6 and Figure 7). These findings revealed that inhibition of cardiac Alox12/15 plus conventional ERT treatment may serve as a beneficial approach for late-onset Fabry patients with cardiomyopathy. Myocardial TGFβ upregulation has been associated with hypertrophy, cardiac remodeling and fibrosis [46], and increased cardiac TIMP-1 expression is closely related to the development of cardiac fibrosis [52]. In the present study, inhibition of Alox12/15 decreased the expression of TGFβ, TIMP-1 and collagen-1, indicating that Alox12/15 may be involved in the complicated network among these fibrosis-related factors. Although a crucial role of Alox12/15 that contributes to the development of cardiac fibrosis in vivo has been reported previously [36], isolated cardiac fibroblasts or patient-specific iPSC-derived cardiac fibroblasts will be required to further elucidate the precise role of Alox12/15 in the fibrosis-related molecular pathways.

The irresponsiveness of FC-iPSC-CMs to α-galactosidase A at late stage of cardiac induction is clinically relevant. It has been shown that cardiomyocyte hypertrophy is not modified in patients receiving ERT for more than three years although the globotriaosylceramide deposits have been cleared [5], while 10 years of ERT effectively decreased left ventricular mass in patients who exhibited no LVH at treatment onset [53]. Our data supported the clinical observations that early treatment of ERT drugs exhibits better efficacy on FC. Importantly, the occurrence of myocardial fibrosis has been known to hinder the treatment efficacy of ERT drugs during the long course of FC [11], suggesting an additional therapeutic regimen such as Alox12/15 pharmacological inhibition is required to improve conventional treatment in patients with poor responses to ERT.

## 4. Materials and Methods

### 4.1. Generation of Patient-Specific iPSCs

Skin fibroblasts from the Fabry patients carrying IVS4+919 G>A mutation were isolated after the patients gave informed consent. All human research was performed in accordance with the review board (IRB) and Committee of Taipei Veterans General Hospital (2013-06-025B). For the generation of iPSCs, the skin fibroblasts were initially cultured in Dulbecco's modified Eagle’s medium (DMEM) supplemented with 10% fetal bovine serum (Life Technologies, Carlsbad, CA, USA), and reprogrammed into iPSCs with the retroviral factors including Oct4, Sox2, Klf4 and Glis1, as reported previously [27]. At least three individual patient-derived iPSC lines were generated per patient and assessed their pluripotency using various tests. Established iPSCs all expressed embryonic stem cell-associated markers including Oct4, Sox2, Nanog, SSEA-3, SSEA-4, Trai-1-60, Trai-81, and were positive stained for alkaline phosphatase staining. Plat-A cells were seeded at 2.5 × 10^6^ cells per 100-mm dish and incubated overnight. Subsequently, using the TransIT^®^-LT1 (Mirus, Madison, WI, USA), 10 μg pMX-containing cDNA was transfected into Plate-A cells culture in 10 mL fresh DMEM. Forty-eight hours after transfection, the virus-containing media was collected and assigned for the infection of target cells. The supernatants containing the four retroviruses were filtered with the 0.45-μm filter and 10 μg/mL polybrene (Sigma-Aldrich, St. Louis, MO, USA) was added. Target cells were seeded into 6-well plates at 5 × 10^4^ cells per well one day before the transduction, and the media was replaced by the virus-containing medium to initiate the transduction. At day 7 post-infection, the transfected cells were passaged and shifted onto the mitotically inactivated MEF feeder layers and further cultured with the iPSC media (DMEM/F12 supplemented with 20% KnockOut serum replacer (KSR; Invitrogen, Carlsbad, CA, USA), 0.1 mM non-essential amino acids (Invitrogen, Carlsbad, CA, USA), 1 mM l-glutamine, 0.1 mM β-mercaptoethanol, 10 ng/mL recombinant human basic fibroblast growth factor (bFGF) and antibiotics (Gibco). To aid the colony formation, 2 μM SB431542 (Stemgent, Cambridge, MA, USA), 0.5 μM PD0325901 (Stemgent), and 0.5 μM thiazovivin were added into the culture medium. These drug-containing media was replaced daily until the detection of iPSC colonies. To prevent the MEF contamination, the iPSCs were transferred to a serum-free and feeder-free culture in HESF V2 medium (Cell Science & Technology Institute, Inc. Tokyo, Japan) without the addition of KSR.

### 4.2. In Vitro Differentiation of iPSCs

The in vitro differentiation potential of iPSCs was evaluated by the ability for EB (embryoid bodies) formation. For the formation of EBs, iPSCs were dispersed into clumps using dispase (Sigma-Aldrich, St. Louis, MO, USA; 1 mg/mL for 30 min) and suspended in ultralow attachment 6-well plates (Corning, Lowell, MA, USA) with DMEM/F12 supplemented with 20% FBS, 0.1 mM NEAA, 1 mM GlutaMax-1, 0.1 mM 2-mercaptoethanol, 50 U/mL penicillin and 50 mg/mL streptomycin. Three days later the aggregated cells were switched onto 0.1% gelatin-coated culture dishes incubating with medium containing FBS. The media was changed every two days. To verify the specific markers for the differentiation into tri-dermal linages, the cells were stained with an anti-α-smooth muscle actin monoclonal antibody (04-1094, Millipore, Burlington, MA, USA), an anti-NF antibody (N1501, Dako, Carpinteria, CA, USA), or an anti-alpha-fetoprotein monoclonal antibody (3903, Cell Signaling, Danvers, MA, USA).

### 4.3. Quantitative PCR

Quantitative RT-PCR was performed as described previously [54]. Briefly, the reverse transcription reactions were carried out using the SuperScript III reverse transcriptase (Invitrogen). The resulting cDNA products of the reverse transcription reactions were assigned for the quantitative PCR that was performed with the Power SYBR Green PCR Master Mix (Applied Biosystems, Foster City, CA, USA), following the manufacturers’ instructions. The primer sequences used for the quantitative PCR are listed in Supplementary Table S1. A 7900HT Fast Real-Time PCR system (Applied Biosystems, Foster City, CA, USA) was used for the detection of the signals yielded by the quantitative PCR. 

### 4.4. Cardiac Differentiation from iPSCs

For the cardiac induction, the differentiation of iPSCs generated either from control subjects or Fabry patients were performed according the established protocol reported previously [31,55]. Briefly, iPSCs were detached using the Accutase solution (Stem Cell Technology, Vancouver, BC, Canada) and were further seeded on the Geltrex-coated plates in the mTeSR1 medium (Stem Cell Technology). The initial culture media was mTeSR1 and RPMI (Life Technologies), supplemented with B-27 or without insulin (Life Technologies) and with the GSK3 inhibitor CHIR99021 (Selleckchem, Houston, TX, USA). After incubation for 24 h, the media was removed and replaced by RPMI/B-27 without insulin. The mixture of old media plus fresh RPMI/B-27 without insulin at a 1:1 ratio was used as the combined media. One day, the media was replaced with combined media containing 5 μM of Wnt signaling inhibitor, IWP2 (Tocris Bioscience, Bristol, UK). At day 5 post-induction, the media was removed and replaced by fresh RPMI/B-27 without insulin again. At day 7 post-induction, RPMI media with B-27 (Life Technologies) was added and changed every three days within the three-week differentiation course. Twenty days after the cardiac induction, the spontaneous beating of embryoid bodies can be obviously detected under light microscopy. Spontaneously beating embryoid bodies were picked up by mechanistic method under dissecting microscope (SZ61, Olympus, Tokyo, Japan) and were then dissected into single cells using Accutase solution (Stem Cell Technology). PSC-Derived Cardiomyocyte Isolation Kit (MACs Miltenyi Biotec, Bergisch Gladbach, Germany) was used to further enrich the cTnT-positive cells. These cells were then plated onto Geltrex-coated dishes for further experiments and analysis.

### 4.5. Western Blot Assay

Protein extraction and Western blot assay were conducted as previously [56]. The lysate proteins were then separated by sodium dodecyl sulfate polyacrylamide gel electrophoresis (SDS-PAGE) using a 12% polyacrylamide gel and electroblotted onto a polyvinylidene difluoride membrane. Subsequently, the non-specific bindings of the membrane were blocked by incubation with 5% nonfat milk for 1 h at room temperature. The membrane was next switched to the TBST buffer containing primary antibodies and 3% nonfat milk at 4 °C overnight, followed by incubation with secondary antibodies conjugated with peroxidase at room temperature for 1 h. The immunoblot signals were developed by an enhanced chemiluminescence system and visualized on the X-ray film. The antibodies used for the Western blot assay are listed in the Supplemental Table S2.

### 4.6. Immunofluorescence Staining and the Quantification of the Cardiomyocyte Size

iPSC-derived cardiomyocytes were initially fixed in the 4% paraformaldehyde solution and permeabilized in 0.1% Triton X-100 solution, followed by the blocking of non-specific binding with the PBS buffer containing 5% normal goat serum. Then, these cells were incubated with primary antibodies using the indicated antibodies and conditions described in Supplemental Table S2. After washing of the cells with PBS for three times, these cells were then incubated with the goat anti-mouse secondary antibodies conjugated with either FITC (green) or PE (red). DAPI (blue) was used for the staining of nucleus. A laser-scanning confocal microscope (Olympus) was used for the imaging of labeled cells and the amount of retained autofluorescent materials were measured in the red (546) and green (488) channels by the quantification of the pixel areas (Adobe Photoshop/Image J software (version 1.50d, National Institutes of Health, Bethesda, MD, USA). The antibodies used for the immunofluorescence staining are listed in the Supplemental Table S2.

For the measurement of cardiomyocyte size, the size was evaluated by measuring the cellular area contents of iPSC-derived cardiomyocytes generated from either normal subjects or patients with Fabry cardiomyopathy. Twenty days after cardiac induction, the spontaneously beating embryoid bodies were dissociated into single cells using Accutase solution (Stem Cell Technology). These cells were then plated onto gelatin-coated dishes for further experiments and analysis. Subsequently, the cellular images of cTnT-positive cells were recorded at 30, 40 and 60 days post-induction using the confocal microscope (FV10i, Olympus). The area of cTnT-positive cells was measured and the cellular area pixels were analyzed using the ImageJ software package (NIH). About fifty cells were analyzed in three independent experiments.

### 4.7. Transmission Electron Microscopy

For the electron microscopic examination of patient’s heart specimen using transmission electron microscopy, heart biopsy specimen from patients with Fabry cardiomyopathy were fixed in 0.1 M cacodylate buffer (pH 7.4) containing 3% glutaraldehyde at 4 °C for 1 h. Subsequently, the samples were then post-fixed with 1% osmium tetroxide (OsO4; pH 7.4). After washing with cold water, the samples were dehydrated in a serial dilution of cold ethanol (from 50% to 100% ethanol, each for 10 min), followed by the infiltration with 100% ethanol/acetone (1:1 mixture) and 100% acetone (each for 15 min). The samples were further infiltrated with 100% acetone/Spurr resin (1:1 mixture and 1:3 mixtures, each for 1 h), then switched to Spurr resin and continuously infiltrated for 24 h, and eventually transferred to a Spurr resin-containing capsule. Polymerization and solidification of Spurr resin was conducted at 72 °C for twenty-eight hours. An ultramicrotome (Leica Ultracut R, Vienna, Austria) was used to trim and cut the resin blocks. After transfer to a 200 mesh copper grids, the sections were stained with 2% uranyl acetate and 2.66% lead citrate, for 20 min and 5 min, respectively. The sections were examined on a JEM1400 electron microscope (JEOL USA, Inc., Peabody, Massachusetts, MA, USA) at 100–120 kV.

### 4.8. LC-MS/MS Analysis

The LC-MS/MS analysis was performed as described previously observed in [57]. Chromatographic separation was performed on a self packed reversed phase C18 nano-column (75 µm I.D. × 200 mm, 2.5 µm, 100 Å) using a mixture of 0.1% formic acid in water and in 80% acetonitrile as mobile phase at 300 nL/min flow rate. The mass survey scan (*m*/*z* range: 200–2000) was performed in an Orbitrap Fusion mass spectrometer (Thermo Fisher Scientific Inc., San Jose, CA, USA) with a mass resolution of 120,000 at *m*/*z* 200. The top twelve most intense ions were sequentially isolated for tandem mass analysis. Protein identification and label-free quantification were accomplished via MaxQuant computational proteomics platform and MaxLFQ software (Max Planck Institute of Biochemistry, Martinsried, Germany. http://www.biochem.mpg.de/5111795/maxquant, MaxLFQ is implemented in the MaxQuant computational proteomics platform). *p* < 0.01 was set as the threshold of significant identification.

### 4.9. Measurement of α-Gal A Enzyme Activity

Ten microliters of cell lysates were mixed with 50 µL of assay buffer (lysis buffer without addition of Triton X-100) supplemented with 4-MUG (6 mM) and *N*-acetyl-d-galactosamine (117 mM) and further incubated at 37 °C for 1 h. The reaction was terminated by adding the Stop solution (0.4 M glycine, pH 10.8; 70 µL). α-Gal A enzyme activity was measured by monitoring the fluorescence (355 nm excitation and 460 nm emission) using the Victor plate reader (Perkin-Elmer, Waltham, MA, USA). Total protein amount was used to normalize the enzyme activity.

### 4.10. ELISA-Based 15(S)-HETE and 12(S)-HETE Measurements

Whole blood samples were collected into 1.8 mg/mL EDTA-K3 tubes and centrifuged at 2500× *g* for 20 min at room temperature to obtain the plasma samples. The aliquots were stored at −80 °C until use. Enzyme-linked immunoassays (ELISAs) were used for 15(S) HETE (Cayman Chemicals, Michigan, MI, USA) and 12(S) HETE (Detroit R&D, Detroit, MI, USA) quantification according to the manufacturers’ instructions (Detroit R&D, Detroit, MI, USA).

### 4.11. Statistical Analysis

The data were expressed as mean ± standard deviation. A statistically significant difference was detected by Student’s *t*-test or one-way ANOVA. For one-way ANOVA, once the difference was detected, a post hoc Tukey test was subsequently performed using SPSS 12.0 (IBM, Armonk, NY, USA). The difference of the gene expression profiles or cardiomyocyte size between control and Fabry cells were analyzed by unpaired Student’s two-tailed *t*-test. The difference was considered to be significant when the *p* value was less than the criterion 0.05.

## 5. Conclusions

Our findings from FC-iPSC-CMs used as in vitro platform indicated the involvement of cardiac Alox12/15 and its secreted metabolites 12(S)-HETE/15(S)-HETE in the pathophysiology of FC with IVS4+919 G>A mutation, particularly for the late phase of disease progression. It will be of great interest whether pharmacological therapeutics, such as targeting cardiac Alox12/15 as an auxiliary intervention, will ameliorate the severity of cellular hypertrophy and fibrotic changes in FC with IVS4+919 G>A mutation, especially in the late stage patients with myocardial-fibrosis and insensitive to ERT.

## Supplementary Materials

Supplementary materials can be found at http://www.mdpi.com/1422-0067/19/5/1480/s1.
