# Supplementary material for: Inhibition of Arachidonate 12/15-Lipoxygenase Improves α-Galactosidase Efficacy in iPSC-Derived Cardiomyocytes from Fabry Patients
Source: Int J Mol Sci. 2018 May 16;19(5):1480. doi: 10.3390/ijms19051480 (PMC5983630; doi:10.3390/ijms19051480)
Supplement: Supplementary file 1 [file ijms-19-01480-s001.pdf]

Supplementary

Inhibition of Arachidonate 12/15-Lipoxygenase Improves α-Galactosidase Efficacy in iPSC-Derived Cardiomyocytes from Fabry Patients

Shih-Jie Chou [1,†], Yueh Chien [1,2,†], Yuh-Lih Chang [1,3], Hsin-Bang Leu [4,5,6], Yi-Ping Yang [2], Ping-Hsing Tsai [2,4], Ying-Hsiu Lai [2], Kuan-Hsuan Chen [3,4], Wei-Chao Chang [7,8,9], Shih-Hsien Sung [4,6] and Wen-Chung Yu [4,6,*]

[1] Institute of Pharmacology, School of Medicine, National Yang-Ming University, Taipei, 11217, Taiwan; ohyeahchou@gmail.com (S.-J.C.) g39005005@gmail.com (Y.C.); ylchang@vghtpe.gov.tw (Y.-L.C.)
[2] Department of Medical Research, Taipei Veterans General Hospital, Taipei, 11217, Taiwan; molly0103@gmail.com (Y.-P.Y.); figatsai@gmail.com (P.-H.T.); d49405004@gmail.com (Y.-H.L.)
[3] Department of Pharmacology, Taipei Veterans General Hospital, Taipei, 11217, Taiwan; sharkshine@gmail.com
[4] Institute of Clinical Medicine, School of Medicine, National Yang-Ming University, Taipei, 11217, Taiwan; hsinbangleu@gmail.com (H.-B.L.); mr.sungsh@gmail.com (S.-H.S.)
[5] Heath Care and Management Center, Taipei Veterans General Hospital, Taipei, 11217, Taiwan
[6] Division of Cardiology, Department of Medicine, Taipei Veterans General Hospital, Taipei, 11217, Taiwan
[7] Center for Molecular Medicine, China Medical University Hospital, Taichung, 40447, Taiwan; T21443@mail.cmuh.org.tw
[8] Graduate Institute of Cancer Biology and Center for Molecular Medicine, China Medical University, Taichung, 40447, Taiwan
[9] Department of Biotechnology, Asia University, Taichung, 41354, Taiwan
* Correspondence: wcyu@vghtpe.gov.tw; Tel.: +886-2-28757394; Fax: +886-2-28757396
† These authors contributed equally to this work.

Supplemental Figure 1

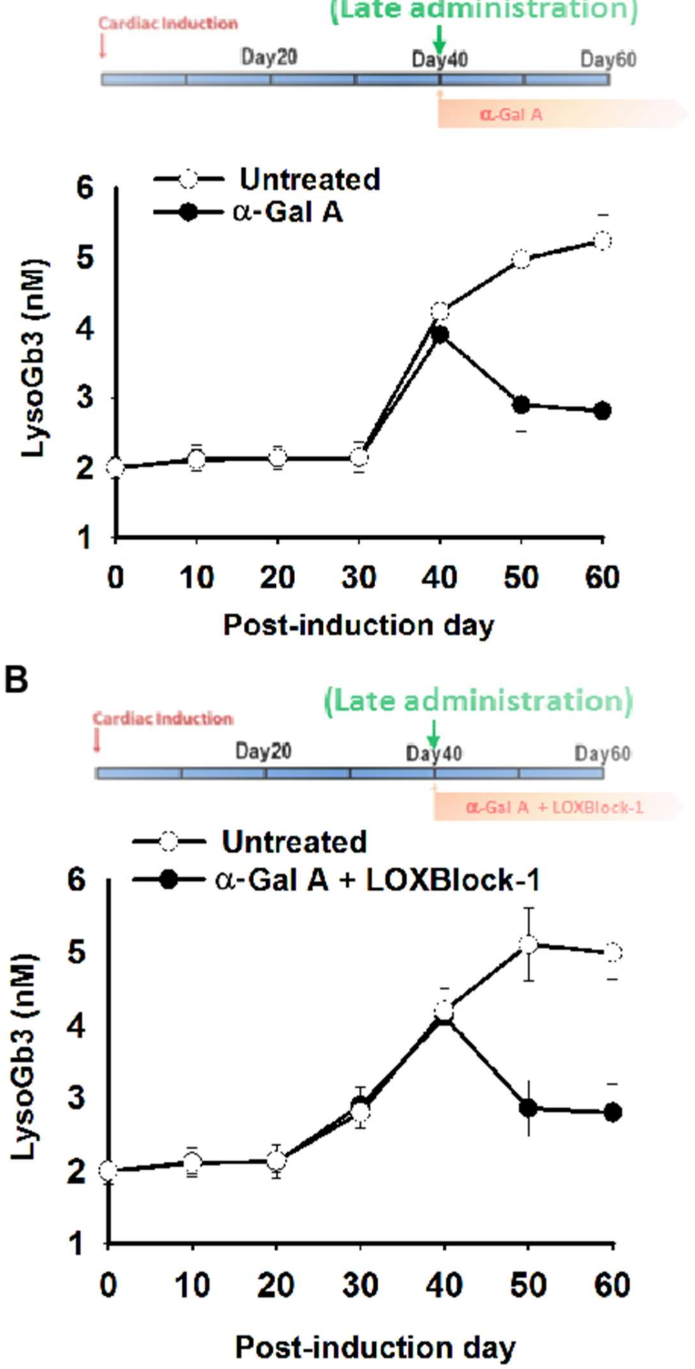

Supplemental Figure 1. Effect of late administration of alpha galactosidase plus Alox12/15 pharmacological inhibitor on lysoGb3 release. LysoGb3 release from FC-iPSC-CMs receiving (A) late-administration of either alpha galactosidase alone or (B) a combination of GLA and LOXBlock-1, was measured.

Supplemental Table S1. List of RT-PCR primers

**The sequences of the primers for quantitative RT-PCR**			
Gene (Accession No.)	Primer Sequence (5' to 3')	Product size (bp)	Tm (°C)
hOCT4 NM_001159542.1	F: CTTCAGGCACTGTGTTCATTG<br>R: TTTGGCTGAACACCTTCCCA	672	60
hSOX2 NM_003106.3	F:GCCCTGCAGTACAACTCCAT<br>R:TTCCTGCAAAGCTCCTACCG	735	60
hNANOG NM_024865.2	F:GAAGACAAGGTCCCGGTCAA<br>R:GGATTCAGCCAGTGTCCAGA	709	60
REX1 NM_174900.3	F:GTGGGCCTTATGTGATGGCT<br>R:TGCGTTAGGATGTGGGCTTT	759	60
hDPPA2 NM_138815.3	F:CCGTCCCCGCAATCTCCTTCCATC<br>R:ATGATGCCAACATGGCTCCCGGTG	606	60
hDppa4 NM_018189.3	F:TAGCACAGCAAAAGAGGCCA<br>R:TGCATGGCCCATAAACAGGT	635	60
hDppa5 NM_001025290.2	F:CGGCACGTAGACATATCCCG<br>R:GGCTTCATTGCATTGGCTGG	366	60
hGDF3 NM_020634.1	F:GTTTGTGTTGCGGTCAGTCC<br>R:TTGGTGGGGATACACACAGC	501	60
hGAPDH NM_002046.5	F:GTCGCCAGCCGAGCCACATC<br>R:CCAGGCGCCCAATACGACCA	83	60
hANF NM_006172.3	F:GCCTAGGGACAGACTGCAAG<br>R:GGCGAGGAAGTCACCATCAA	170	60
hACTA1 NM_001100.3	F:CAGGGCGTCATGGTCGGT<br>R:GTGCCAGATCTTCTCCATGTCATC	141	60
hMYL2 NM_000432.3	F:CTCATCTCTCTCCCCCGAGT<br>R:TGGAACATGGCCTCTGGATG	161	60
hMYL7 NM_021223.2	F:GGAGTTCAAAGAAGCCTTCAGC<br>R:CTCCTCTGGGACACTCACCT	112	60

Supplemental Table S2. List of proteins tested by antibodies

Protein	Assay	Antibody	Origin	Dilution	Incubation period
GAPDH	WB	rpab	Ab9385, Abcam, Inc	1:5000	60 min
OCT3/4	IF	rpab	#2840, Cell Signaling, Inc	1:400	O.N.
NANOG	IF	rpab	#3580, Cell Signaling, Inc	1:800	O.N.
TRA 1-60	IF	mmab	#4746, Cell signaling, Inc	1:1000	O.N.
TRA 1-81	IF	mmab	#4745, Cell Signaling, Inc	1:1000	O.N.
SMA	IF	rpab	Ab52218, Abcam, Inc	1:500	O.N.
NF	IF	mmab	MAB1615, Millipore, Inc	1:200	O.N.
AFP	IF	mmab	#3903, Cell Signaling, Inc	1:100	O.N
HNF3β	IF	rpab	Sc-20692, Santa Cruz, Inc	1:200	O.N.
NESTIN	IF	rpab	ABD69, Millipore, Inc	1:500	O.N.
α-ACTININ	IF	mmab	Ab9465, Abcam, Inc	1:200	O.N.
MYL2	IF	rpab	Ab79935, Abcam, Inc	1:200	O.N.
MYL7	IF	mmab	Ab68086, Abcam, Inc	1:200	O.N.
cTNT	IF	mmab	Ab10214, Abcam, Inc	1:200	O.N.
GLA	WB	rpab	GTX101178, GeneTex, Inc	1:1000	60 min
Crystallin A3	WB	rpab	GTX109207, GeneTex, Inc	1:1000	60 min
Crystallin A4	WB	rpab	GTX109526, GeneTex, Inc	1:1000	60 min
ALOX12	WB, IF	rpab	PA5-26020, Thermo, Inc	1:1000, 1:200	60 min, O.N.
ALOX15	WB, IF	mmab	Ab119774, Abcam, Inc	1:200	60 min, O.N.
α-TUBULIN	IF	rpab	#5568, Cell Signaling, Inc	1:10000	60 min

Abbreviations: WB, Western blot; mmab, mouse monoclonal antibody; rpab, rabbit polyclonal antibody; IF, immunofluorescence; O.N.: over night.
